# Automated Single-Cell Analysis in the Liquid Biopsy of Breast Cancer

**DOI:** 10.3390/cancers17172779

**Published:** 2025-08-26

**Authors:** Stephanie N. Shishido, George Courcoubetis, Peter Kuhn, Jeremy Mason

**Affiliations:** 1 Convergent Science Institute in Cancer, Michelson Center for Convergent Bioscience, University of Southern California, Los Angeles, CA 90089, USA; sshishid@usc.edu (S.N.S.); pkuhn@usc.edu (P.K.); 2 Catherine and Joseph Aresty Department of Urology, Institute of Urology, Keck School of Medicine, University of Southern California, Los Angeles, CA 90033, USA; 3 Norris Comprehensive Cancer Center, Keck School of Medicine, University of Southern California, Los Angeles, CA 90033, USA; 4 Department of Biological Sciences, Dornsife College of Letters, Arts, and Sciences, University of Southern California, Los Angeles, CA 90089, USA; 5 Department of Aerospace and Mechanical Engineering, Viterbi School of Engineering, University of Southern California, Los Angeles, CA 90089, USA; 6 Department of Biomedical Engineering, Viterbi School of Engineering, University of Southern California, Los Angeles, CA 90089, USA

**Keywords:** breast cancer, liquid biopsy, peripheral blood, fluorescent whole-slide imaging, circulating tumor cell, screening, mathematical modeling, automation

## Abstract

**Background/Objectives**: Breast cancer (BC) is the most prevalent cancer worldwide, with approximately 40% of early-stage BC patients developing recurrence despite initial treatments. Current diagnostic methods, such as mammography and solid tissue biopsies, face limitations in sensitivity, accessibility, and the ability to characterize tumor heterogeneity or monitor systemic disease progression. **Methods**: To address these gaps, this study investigates a fully automated analysis workflow using data derived from fluorescent Whole-Slide Imaging (fWSI) for detecting and classifying rare cells (circulating tumor and tumor microenvironment cells) in peripheral blood samples. Our methodology integrates supervised machine learning algorithms for rare event detection, immunofluorescence-based classification, and statistical quantification of cellular features. **Results**: Using a fWSI dataset of 534 cancer and non-cancer peripheral blood samples, the automated model demonstrated high concordance with manual annotation, achieving up to 98.9% accuracy and a precision-sensitivity AUC of 83.2%. Morphometric analysis of rare cells identified significant differences between normal donors, early-stage BC, and late-stage BC cohorts, with distinct clusters emerging in late-stage BC. **Conclusions**: These findings highlight the potential of liquid biopsy and algorithmic approaches for improving BC diagnostics and staging, offering a scalable, minimally invasive solution to enhance clinical decision-making. Future work aims to refine the automated framework to minimize errors and improve the robustness across diverse cohorts.

## Simple Summary

Breast cancer (BC) diagnostic methods, such as mammography and tissue biopsy, have inherent limitations in both accuracy and accessibility. A standard blood draw allows for the detection of rare events, such as circulating tumor cells (CTCs), that can be indicative of both cancer and the extent of the disease within the body. This study demonstrates the potential of a fully automated, liquid biopsy workflow as a highly scalable and minimally invasive companion to current methods that can detect rare events useful for identifying and characterizing BC within an individual.

## 1. Introduction

Breast cancer (BC), with 7.8 million global cases diagnosed in the past 5 years, is the most common cancer in women and the most prevalent overall [1,2,3]. Most women are diagnosed with early-stage BC (94%), without evidence of widespread disease; however, despite this and with the administration of subsequent treatments, 40% of these patients will experience recurrence in their lifetime [4,5,6,7,8,9]. Late-stage BC (i.e., relapse, progression, and onset of distant metastasis) has a 5-year survival rate of less than 30%, significantly lower than that of early-stage BC at 91% [1,3]. Given this, it is vital that screening methods be improved, and that robust stratification of early-stage BC be made possible at the time of the initial diagnostic workup.

Currently, the standard screening method for BC is mammography, with a tissue biopsy to confirm diagnosis [3,4]. Despite mammography being common practice, only about 60% of all cases are currently diagnosed via the screening pathway. This is primarily due to two factors, one being the limited (86.9%) sensitivity of mammography, and second, its limited use across the at-risk patient population, with only 76.4% of women aged 50–74 years having regularly scheduled mammograms every two years if at average risk for BC [10,11,12,13]. In addition, only limited screening pathways exist for younger patients. For patients diagnosed with BC, the extent of tumor burden and prediction of treatment response are assessed via imaging and clinical evaluation of symptoms [4]. To identify the spread of disease, cross-sectional advanced imaging is sometimes used; however, it is often inconclusive, expensive, and unable to provide deeper insights into the status of the molecular tumor profile.

Solid tissue biopsies are widely used in current clinical care as they contain a wealth of information. They can provide information on molecular profiles, histological subtyping, and tumor biomarkers. They can also lend advice on treatment planning. However, several caveats persist. First, it is not always easy or straightforward to access and biopsy the primary tumor or metastatic lesions. Second, these biopsies are designed to survey a precise sampling area and may fail to capture the true heterogeneity of the tumor [14,15,16,17,18]. Circulating tumor cells (CTCs) have the potential to resolve the spatial heterogeneity problem, as they have been shown to shed from primary and metastatic tumor sites [19,20,21,22,23,24,25]. Third, solid biopsies will inherently fail to characterize subclinical systemic disease spread. And lastly, they are infeasible for longitudinal monitoring due to their invasiveness and both pain and potential risk that are inflicted on the patient [26,27,28,29,30].

Consequently, better screening methods accessible to all individuals and more precise staging at diagnosis and prognosis are essential components for improving clinical management of BC patients. To evaluate the potential of a liquid biopsy for early BC detection and assessment, in a previous study, we utilized a customized fluorescent assay on whole-slide cell monolayer preparations to identify and analyze rare cells, such as CTCs, in peripheral blood (PB) samples [31]. We revealed statistically significant differences in circulating rare cell populations, including CTCs, through analyzing a cohort of early-stage BC patients, late-stage BC patients, and age- and gender-matched controls through a labor-intensive manual process [31]. Patient-level modeling to predict Cancer vs. Normal and Early-stage vs. Late-stage achieved Area Under the Curve (AUC) values of 0.99 and 0.91, respectively, indicating 99% accuracy in differentiating cancerous from non-cancerous samples and 91% accuracy in stratifying disease stage. While these results were obtained through a manual approach supported by computation, these imaging datasets require extensive human interpretation. We hypothesize that an automated data science approach could aid in this analysis and accelerate the discovery process.

In this study, we developed a fully automated methodology to investigate the scalability of a liquid biopsy test using an expanded validation set. The approach incorporates supervised machine learning algorithms for rare event identification, event classification based on IF expression, and quantification of results. Our automated system generates cell-level prediction and classification models that detect and assign IF phenotypes to rare events. The results highlight the potential of algorithmic approaches to enhance annotation efficiency and accelerate the discovery phase of liquid biopsy analyses, while emphasizing the need for manual intervention to minimize errors.

## 2. Materials and Methods

### 2.1. Study Design, Patient Information

A total of 1070 deidentified fluorescent whole-slide images (fWSIs) from 534 PB samples corresponding to a variety of human samples (carcinoma patients and non-cancerous normal donors [NDs]) were utilized in designing the automated approach (Design Set). The Design Set was split into 1030 fWSIs for training the model and 40 fWSIs for testing. To focus on an application in BC, the test set of fWSIs was only comprised of BC samples. An additional 779 fWSIs from 410 PB samples were utilized in the application and assessment of the automated approach (Apply Set). Samples were previously published: BC (n = 575) [31,32], pancreatic cancer (n = 123) [33], upper tract urothelial carcinoma (n = 51) [34,35], bladder cancer (n = 50) [36], lung cancer (n = 40) [37], colorectal cancer (n = 18) [38], and NDs (n = 87) [31,33,39]. Patient recruitment occurred according to Institutional Review Board-approved protocols, with all participants providing written informed consent. All samples were collected between 5 April 2013 and 22 September 2022.

### 2.2. LBx Acquisition, Processing, and Cryobanking

The fWSI workflow we utilize here is aimed at identification and characterization of analytes from liquid biopsy samples. Approximately 7.5 mL of PB was collected in 10 mL Cell-free DNA blood collection tubes (Streck, Omaha, NE, USA) at the clinical site and shipped to our laboratory for processing within 48 h as previously described [36,40]. In brief, the samples were subjected to red blood cell lysis in isotonic ammonium chloride solution. All nucleated cells were plated as a monolayer on custom glass slides (Marienfeld, Lauda, Baden-Württemberg, Germany) with approximately 3M cells per slide. The cells were then blocked with 7% bovine serum albumin (BSA), dried, and cryopreserved at −80 °C until analysis. Each sample yields approximately 14 prepared glass slides, resulting in ~0.5 mL of PB plated on each.

### 2.3. Staining, Scanning, and Pre-Processing

Prior to IF staining via an IntelliPATH FLX autostainer (Biocare Medical LLC, Pacheco, CA, USA), 2 glass slides per PB sample were thawed for 1 h at room temperature and subsequently fixed using 2% paraformaldehyde (PFA) for 20 min. We then used 10% goat serum (Millipore, Billerica, MA, USA) for 20 min to block nonspecific binding sites. The specific IF antibodies utilized in the customized fluorescent assay are DAPI (D; nuclear identification), cytokeratin (CK; epithelial cells), CD45 (white blood cells), CD31 (endothelial cells), and Vimentin (V; mesenchymal cells). CD45 and CD31 are visualized in the same IF channel (CD). Specifically, the following steps were applied to each slide as detailed previously [40]:

- Incubated for 4 h with a conjugate containing the following:
  ○ 2.5 μg/mL of a mouse IgG1 anti-human CD31:Alexa Fluor 647 mAb (clone: WM59, MCA1738A647, BioRad, Hercules, CA, USA);
  ○ 100 μg/mL of a goat antimouse IgG monoclonal Fab fragments (115–007–003, Jackson ImmunoResearch, West Grove, PA, USA).
- Cold methanol used for 5 min to permeabilize the cells.
- Incubated for 2 h with an antibody cocktail consisting of the following:
  ○ mouse IgG1/IgG2a anti-human CK 1, 4, 5, 6, 8, 10, 13, 18, and 19 (clones: C-11, PCK-26, CY-90, KS-1A3, M20, A53-B/A2, C2562, Sigma, St. Louis, MO, USA);
  ○ mouse IgG1 anti-human CK 19 (clone: RCK108, GA61561–2, Dako, Carpinteria, CA, USA);
  ○ mouse antihuman CD45:Alexa Fluor 647 (clone: F10–89–4, MCA87A647, AbD Serotec, Raleigh, NC, USA);
  ○ rabbit IgG antihuman V: Alexa Fluor 488 (clone: D21H3, 9854BC, Cell Signaling Technology, Danvers, MA, USA).
- Incubated for 1 h with Alexa Fluor 555 goat anti-mouse IgG1 antibody (A21127, Invitrogen, Carlsbad, CA, USA) and 4′,6-diamidino-2-phenylindole (DAPI; D1306, Thermo Fisher Scientific, Waltham, MA, USA).
- Mounted with a glycerol-based aqueous mounting media.
- Coverslipped to maintain cell integrity.

After IF staining, slides were scanned at 100× magnification in each of the IF channels as previously performed [41]. Given the staining assay used for these data, the scanning process produces 9216 total frames of view (i.e., 2304 frames × 4 IF channels) to comprise a single fWSI. The EBImage package in R (version 4.22.1) is used to mask each cellular (DAPI+) event and subsequently generate morphological features. With each of the IF channels and paired combinations (e.g., CK + V), each event produces 761 descriptive features. Examples include eccentricity, area, mean radius, major axis, perimeter, and channel intensity.

### 2.4. Rare Event Detection, Identification, Classification, and Enumeration

To detect rare events, we used our previously reported framework of OCULAR (Outlier Clustering Unsupervised Learning Automated Report) [36,40]. This methodology uses principal component analysis (PCA) to reduce the 761 descriptive features to 350 principal components. It then utilizes the reduced dimension space and hierarchical clustering to identify clusters with a small number of cells as well as individual cells that are distinctly different from the median cell (i.e., large computational distance). For further discrimination, and filtering out technical artifacts, we developed a machine learning classification model to predict whether an event is interesting (i.e., biologically relevant) or not. The positive class consisted of previously identified rare events that were deemed to be biologically relevant to the disease under study. The negative class consisted of previously identified rare events that were deemed to be not biologically relevant (e.g., technical artifacts). The events in both classes were categorized as such due to a multitude of reasons including their shape, fluorescent intensities, localizations of signals within the segmented area, as well as the characteristics of the neighboring events. We utilized a histogram gradient boosting algorithm [42] with k-fold cross-validation [43,44] over 1000 iterations to select the best performing models. We also employed hyperparameter grid searches [45] on the morphometric features to identify the subsets that yielded the best accuracy. The individual prediction confidences of each event were used as cutpoints to evaluate the model and balance sensitivity (i.e., correctly identifying interesting events) with specificity (i.e., correctly identifying non-interesting events).

Additionally, we utilized a trio of machine learning classification models (random forest architectures) to predict the CK, V, and CD channels to classify the rare, interesting cells by their IF expression level [33,34,39]. With these, we stratified the identified cells into 8 distinct types and subsequently enumerated each category for a given sample. We normalized these values based on the amount of blood analyzed using the total number of cells detected and the automatically determined white blood cell counts for the sample, allowing for fractional counts of cells/mL.

### 2.5. Cellular Morphometric Comparison and Statistical Analysis

We investigated the differences at the liquid biopsy level between NDs, early-stage BC patients, and late-stage BC patients via cell-level enumerations. First, we created Uniform Manifold Approximation and Projection (UMAP) plots of the predicted interesting, rare events based on a subset of the 761 morphometric features, color-coded by their predicted channel type. We then enumerated the 8 cell types across the cohorts and statistically compared them using the Mann–Whitney U Test, with statistical significance set at 0.05.

## 3. Results

### 3.1. Development of Automated Rare Cell Stratification Model

The 1070 fWSIs to be used in the training and testing sets were first applied to the OCULAR framework to reduce the ~2 billion cells to ~1 to 2 million rare events. This subset is comprised of ~50,000 biologically relevant cells (positive class) and the remaining events that are deemed as not interesting (negative class), both confirmed by manual review. The performance of the rare cell identification model was evaluated based on concordance with manual analyst annotations, with metrics provided in Table 1. Overall, the model demonstrated high accuracy, ranging from 96.5% at a 50% confidence threshold to 98.9% at a 90% threshold. Increasing the confidence threshold improved precision (from 37.1% at 50% to 68.4% at 90%) and specificity (from 96.4% to 99.1%) but reduced sensitivity (from 97.6% to 85.5%). As confidence thresholds increased, the model predicted fewer non-interesting events (average of 97.6 at 50% vs. 23.2 at 90%), but this resulted in more true positives being missed (average of 1.4 missed events at 50% vs. 8.6 at 90%). Notably, many of the false positives were attributed to biologically irrelevant artifacts such as bubbles and fluorescent flares. These findings underscore the adaptability of the model to different application contexts, where confidence thresholding can be tuned to prioritize either sensitivity or precision depending on experimental needs.

### 3.2. Morphometric Analysis

**Figure 1:**
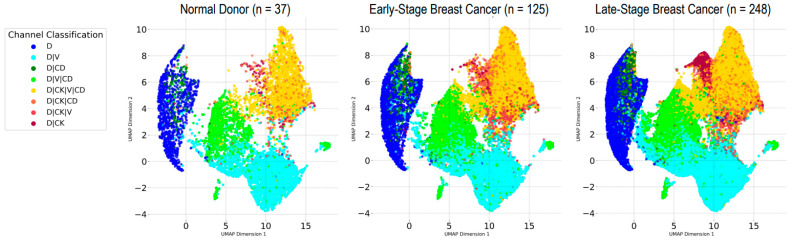
UMAP visualization of rare cell morphometrics for each cohort color coded by channel-type classification. Generated using cellular and nuclear area and mean channel intensity for each fluorescent channel.

For downstream investigation, the automated rare cell identification model at the 50% threshold was applied to the Apply Set, which consisted of 779 fWSIs from non-cancer NDs (n = 74; samples = 37), early-stage BC (n = 248; samples = 125), and late-stage BC (n = 457; n = 248). These were excluded from training and testing to validate the approach. To compare the rare cells detected, UMAP visualizations were generated as a dimensionality reduction technique projecting the high-dimensional data onto a low-dimensional space while preserving the local and global structure of the data (Figure 1). UMAP analysis confirms overlap in the rare cell phenotypes identified by the assay in ND, early-stage BC, and late-stage BC cohorts, as well as distinct signatures relative to each sample source. This validates that both the assay is consistently labeling and that the algorithm is consistently identifying the rare (outlier) events in any sample, regardless of origin. Across all cohorts, the DAPI only cells form a distinct cluster, although it exhibits some heterogeneity. The remaining three major clusters observed are primarily composed of D|V, D|V|CD, and D|CK|V|CD cells. Interestingly, a well-defined cluster emerges specifically in the late-stage BC cohort for D|CK cells. This is consistent with previous findings, which found enumerations of D|CK to be significantly higher in late-stage BC cohorts compared to ND or early-stage BC cohorts.

### 3.3. Cohort Level Analysis

**Figure 2:**
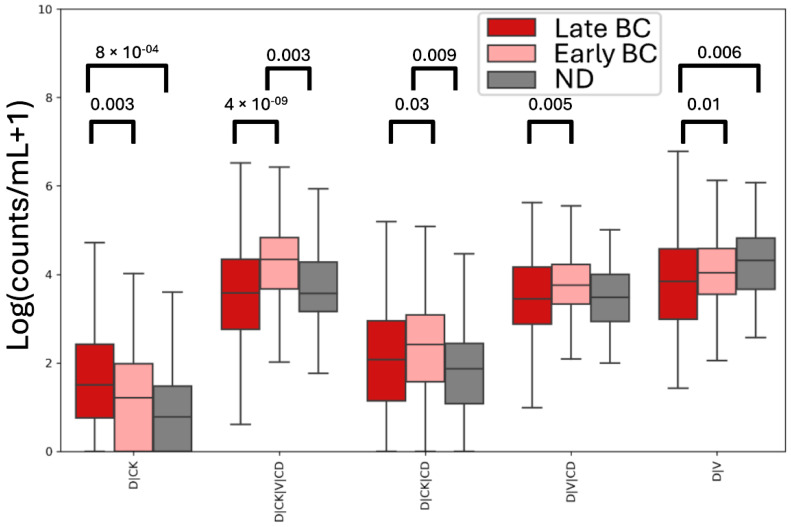
Cohort level analysis of rare cells using the automated approach. Box plot analysis of channel-type rare cell enumerations (log counts) detected in each cohort. Pairwise comparison of each predicted cell type was statistically compared via Mann–Whitney U test.

Rare cells detected in each cohort were normalized to count/mL for comparison (Figure 2). There is an observable statistically significant difference between ND and early-stage BC for D|CK|V|CD (*p*-value = 0.003) and D|CK|CD (*p*-value = 0.009) rare cells, and between ND and late-stage BC for D|CK (*p*-value = 8 × 10^−4^) and D|V (*p*-value = 0.006) rare cells. Additionally, there is a significant difference between early-stage and late-stage rare cell counts for D|CK (*p*-value = 0.003), D|CK|V|CD (*p*-value = 4 × 10^−9^), D|CK|CD (*p*-value = 0.03), D|V|CD (*p*-value = 0.005), and D|V (*p*-value = 0.01). There were no significant differences measured in the D|CD, D|CK|V, and D rare cell groups across any of the cohorts.

## 4. Discussion

This study highlights the ability of automated analysis of single-cell data derived from fWSI data as a liquid biopsy approach to effectively identify and classify rare circulating cells from PB samples without prior enrichment. Using machine learning algorithms and five biomarkers, the platform identified and characterized circulating cells of epithelial, mesenchymal, endothelial, and hematologic origins. Importantly, the study observed a statistically significant increase in CTCs in late-stage BC compared to early-stage, consistent with prior research [31] linking higher CTC counts to metastatic dissemination [46]. This is expected given that these late-stage patients have active disease that is metastasizing via the circulatory system and therefore would yield increased activity within the blood. Additionally, specific phenotypic subtypes of rare circulating cells, with morphology consistent for CTCs [31,40,47] and endothelial cells [38,48], were automatically detected and phenotyped, underscoring the heterogeneity of circulating tumor-related analytes. These findings demonstrate the feasibility of a fully automated, non-enrichment liquid biopsy approach to provide robust diagnostic and prognostic insights.

Identification of rare cells from fWSI datasets typically requires extensive human interpretation, performed by a pathologist-trained technician supported by computational algorithms as previously described, which restricts their scalability. The fully automated approach developed here addresses these limitations by enhancing the identification of rare cells and subsequent phenotyping using advanced computational methods toward an operator-independent solution by leveraging the substantial human-annotated data we have curated.

We have previously revealed profound heterogeneity and plasticity among CTCs, reflecting cellular plasticity and underscoring the need for single-cell analyses in understanding cancer progression [40,47,49]. These insights point to the need for progress in developing robust, automated, and efficient methodologies for detecting rare cells and phenotyping them once identified. Automated analysis for rare cell detection is crucial for advancing precision medicine due to its scalability, efficiency, and accuracy. Manual analysis, while valuable, is labor-intensive and prone to human error, limiting its feasibility for large-scale studies or clinical applications. Automation enables the rapid processing of vast datasets, facilitating the detection of rare cellular events with higher consistency and reproducibility. This speed and scalability are essential for timely decision-making in diagnostics, monitoring treatment efficacy, and conducting large population-based studies. Furthermore, automated systems can integrate complex algorithms to identify subtle patterns in data that may be overlooked by manual methods, improving sensitivity and specificity. By reducing the reliance on skilled personnel for routine tasks, automation also lowers costs and makes cutting-edge technologies accessible to more laboratories and clinics, ultimately accelerating research and improving patient care.

The concept of automated cell identification and classification is not a novel concept within the field of fluorescence microscopy. In fact, many other groups have investigated this application as it relates to their workflows [50,51,52]; however, each contains specific limitations that are not observed in our approach. Namely, they rely on a secondary data collection (e.g., scRNA-seq data), they are designed for the identification of a single phenotype, or they only consider cancer cells vs. non-cancer cells (i.e., they ignore tumor microenvironment cells). Our platform is designed to work with fWSIs alone to generate the relevant morphometric parameters to identify every cell of interest that would be tumor-related. Given this realization, we have also replicated this framework with a secondary immunofluorescence assay designed for the identification and monitoring of multiple myeloma cells on fWSIs.

While these automated tools boast many advantages within the field of biological sciences, they are not without their challenges. Although the cell-level models described here to identify and classify biologically relevant events perform exceptionally well, they do suffer from a quantifiable and consistent level of error in incorrectly identifying rare events. Additionally, the accuracy is directly linked to the training data used to build the models. In essence, if a new and relevant biological cell type is presented for identification and classification, there is a chance that the model incorrectly predicts it and filters all of these into the negative class, potentially overlooking a significant biomarker for a subset of patients. It is also important to understand how the challenges and errors at the cell-level extrapolate up to patient-level models. The cellular landscape reflects the presence or absence of disease as well as the diversity of the individual. Significant shifts in this landscape can mean the difference between unnecessary medical tests and missed diagnoses. Thus, it is highly critical to maximize both sensitivity and specificity at all levels of model building to ensure maximum benefit for the patients. At the cell level, we addressed this by including samples from a variety of different cancer types in our training data. In doing so, we were able to maximize the number of correctly identified biologically relevant cells (sensitivity) while simultaneously minimizing the amount of junk that was missed (specificity).

In short, automation is a game-changer in the healthcare setting, transforming how diagnostic tests are carried out, impacting efficiency and accuracy. The integration of automated processes has streamlined analyses, resulting in quicker turnaround times, increased throughput, and improved precision in testing [53]. Manual input can be time-consuming with a risk of error that may compromise outcomes [54,55], especially in the healthcare environment. AI algorithms can analyze datasets quickly with precision to interpret results for diagnostic accuracy [56]. It is important to approach automated implementation strategies with caution and acknowledge the challenges.

## 5. Conclusions

This study demonstrates the capabilities of automating and scaling a liquid biopsy framework for the detection and classification of rare cells from a PB sample, enabling earlier detection, enhanced accessibility, and improved patient outcomes. Herein, we find that the rare cell profile automatically identified and classified is heterogeneous and distinct in patients with BC and in NDs. While the results are promising, additional studies are warranted to investigate the liquid biopsy signal within specific cohorts as well as those with benign, non-cancerous conditions.

## Figures and Tables

**Table 1:** Performance metrics for the automated rare cell identification model at various confidence thresholds.

Confidence Thresholds	50%	60%	70%	80%	90%
Accuracy (%)	96.5	97.1	97.7	98.3	98.9
Precision (%)	37.1	42.0	47.7	55.7	68.4
Sensitivity (%)	97.6	96.6	95.4	92.8	85.5
Specificity (%)	96.4	97.1	97.7	98.4	99.1
Average False Negative (Rare Events Missed)	1.4	2.0	2.7	4.2	8.6
Average False Positive (Common Predicted Rare)	97.6	78.9	61.7	43.6	23.2

## Data Availability

All data discussed in this manuscript are included in the main manuscript text. The imaging data are available through the BloodPAC Data Commons utilized for the previous publications. The code for training the machine learning models is available for download on GitHub (https://github.com/CSI-Cancer/ocular_streamlining).

## Abbreviations

The following abbreviations are used in this manuscript:

BC	Breast cancer
CTC	Circulating tumor cell
PB	Peripheral blood
IF	Immunofluorescence
AUC	Area under the curve
DAPI	4′,6-diamidino-2-phenylindole
D	DAPI
CK	Cytokeratin
V	Vimentin
CD	CD45/CD31
OCULAR	Outlier Clustering Unsupervised Learning Automated Report
PCA	Principal component analysis
ND	Normal donor
UMAP	Uniform Manifold Approximation and Projection
BSA	Bovine Serum Albumin
fWSI	Fluorescent Whole Slide Images
